# Aspirin Sensitivity and Chronic Rhinosinusitis with Polyps: A Fatal Combination

**DOI:** 10.1155/2012/817910

**Published:** 2012-08-14

**Authors:** Hendrik Graefe, Christina Roebke, Dirk Schäfer, Jens Eduard Meyer

**Affiliations:** ^1^Departments of Otorhinolaryngology, Head and Neck Surgery, and Plastic Surgery, Asklepios Medical School, Lohmuehlenstraß 5, 20099 Hamburg, Germany; ^2^Allergy and Intolerance Laboratory, Medical Clinic III, Friedrich-Alexander-University Erlangen-Nuremberg, Glückstraße 4a, 91054 Erlangen, Germany

## Abstract

Aspirin-exacerbated respiratory disease (AERD) refers to aspirin sensitivity, chronic rhinosinusitis (CRS), nasal polyposis, asthma, eosinophil inflammation in the upper and lower airways, urticaria, angioedema, and anaphylaxis following the ingestion of NSAIDs. Epidemiologic and pathophysiological links between these diseases are established. The precise pathogenesis remains less defined, even though there is some progress in the understanding of several molecular mechanisms. Nevertheless, these combinations of diseases in patients classified by AERD constitute a fatal combination and may be difficult to treat with standard medical and surgical interventions. This paper reviews in brief the epidemiology, clinical features, diagnosis, molecular pathogenesis, and specific therapies of patients classified by AERD and postulates future attempts to gain new insights into this disease.

## 1. Introduction

Patients suffering from nasal polyps remain one of the more challenging groups of patients to manage. Unfortunately, the precise pathogenesis of nasal polyp formation remains poorly defined. However, inflammation of the upper and lower airways is well documented, and epidemiologic and pathophysiological links between chronic rhinosinusitis (CRS) without or with nasal polyps, asthma, and/or eosinophilic inflammation have been established by recent investigation [1–3]. The association of nasal polyps, asthma, and hypersensitivity to aspirin was first described by Widal et.al in 1922 [4] and thereafter popularized by Samter and Beers in 1968 thoroughly characterizing the clinical picture [5]. This syndrome has been termed “Syndrome de Widal” or “Samter's Triad.” Severe cutaneous and systemic adverse reactions upon ingestion of “aspirin” were first documented in 1902 by Hirschberg [6], shortly after the market launch of aspirin. The diverse terms used in medical literature describing the adverse reactions upon ingestion of nonsteroidal anti-inflammatory drugs (NSAIDs) had been recently reviewed and summarized [7].

This subset of patients with recurrent nasal polyps, asthma, and NSAIDs remains one of the more challenging groups of patients. The term aspirin-exacerbated respiratory disease (AERD) refers to the clinical syndromes of chronic rhinosinusitis (CRS), nasal polyps, bronchoconstriction in asthmatics, and/or eosinophil inflammation in the upper and lower airways, urticaria, angioedema, and anaphyalxis following the ingestion of NSAIDs blocking the COX-1 enzyme. In this concern, NSAIDs are an exacerbating factor rather than an underlying disease. This classification system was proposed by Stevenson et al. in 2001 [8] allowing a better understanding, which type or clinical reactions constitute the subject of the publication. AERD comprises the description of physical reactions, underlying airway-related diseases, and inhibitors of cyclooxygenase (COX). AERD is subdivided, based on physical reactions, to (1) NSAID-induced rhinitis and asthma, (2) NSAID-induced urticaria/angioedema, (3) multiple-drug-induced urticaria/angioedema, (4) single-drug-induce anaphylaxis, and (5) single-drug- or NSAID-induced blended reaction; by definition, there are none underlying diseases concerning the subclassification (3) to (5).

The appearance of diseases mentioned above in combination with the intake of NSAIDs constitutes a fatal combination for some patients. Therefore, current epidemiology, clinical features, diagnostic approaches, molecular pathogenesis, and AERD specific therapies will be elaborated and postulates of future attempts to gain new insights into this disease will be presented.

## 2. Epidemiology

*AERD* has been estimated to affect 0.3 to 2.5% of the general population [2, 8, 9]. The frequency of symptoms associated with AERD published in literature is 5–10% with rhinitis, 5–30% with nasal polyps, 10% with bronchial asthma, 25–30% with nasal polyps and bronchial asthma, and 5–10% with urticaria/Quincke's edema [1, 2, 9–13]. The estimation of prevalence of AERD varies depending on the determination through questionnaire (11–20%), medical record (~3%), or oral provocation test (21%) [2]. Therefore, AERD might be over- as well as underdiagnosed depending on the diagnostic tool used. The onset of AERD is typically during the third decade and is more commonly reported in females (~3 : 2) [14, 15]. Ethnic preferences are not described and only rare familial associations were mentioned [2, 9–12].

*CRS* is estimated as the most frequent chronic diseases worldwide with an intense impact on healthcare system and on the quality of life of patients [15]. More than 30 million Americans are involved [16] causing over 6 billion US $ burden for the health care system worldwide [17]. The prevalence of CRS is difficult to estimate due to different diagnostic criteria, heterogenous group of patients, treatment by different medical professions, and inconsistent definitions but is assumed to reveal 5% ranging from 1 to 19%. Up to 70% of patients with CRS also suffer from asthma and aspirin sensitivity [14, 18–23]. The incidence and prevalence of *CRS with nasal polyps(CRSwNP)* is estimated with 2–4% [14, 18–24] and 31–60% [13, 25, 26], respectively. CRSwNP was observed in 7% of asthmatics but rises to 15% of patients with CRSwNP and aspirin sensitivity [13, 27, 28].

These tremendous discrepancies of aspirin-sensitive patients who suffer from nasal polyps have most likely been caused by the diagnostic techniques which have been used.

## 3. Clinical Features

In patients classified by AERD rhinitis appears first during the third decade with concomitant onset of nasal congestion, hyposmia, chronic rhinorrhea, and progress to chronic pansinusitis followed by nasal polyps which frequently relapses after surgery. Finally, the disease results in NSAID-triggered hypersensitivity of the lower airways with the symptoms of asthma. About fifty percent of the patients demonstrate chronic, severe, corticoid-dependent asthma, often accompanied by systemic anaphylactoid reactions. Based on our recent knowledge, once appeared, AERD remains throughout life, even though sporadic disappearance of intolerance has been reported [1–3, 29–31].

Although there are typical clinical features, AERD is most likely underdiagnosed as exemplified above. Atopy is present in approximately 30% of patients classified by AERD, which was significantly higher in patients with positive rather than negative skin tests [32].

The formation of nasal polyps in patients suffering from AERD follows an aggressive course filling the nasal cavity, often protruding anteriorly in the face or posteriorly into the nasopharynx. Facial deformation is common, due to midfacial expansion, which occurs as a consequence of the increased pressure on the bones from nasal polyps [32, 33]. A strong positive correlation has also been found between the number of polypectomies and the peripheral blood eosinophil count [34, 35].

## 4. Diagnostic Approaches

The diagnosis of AERD is a major challenge not only in patients suffering from CRS with/without nasal polyps and/or bronchial asthma, but also in individuals without known underlying airway-related diseases [1–3]. The diagnostic approach of AERD is based on the clinical picture as outlined above. This will be supported in preposition by imaging techniques, including computed tomography or endoscopy. Some patients have a definitive history of adverse reactions to NSAIDs. However, many patients also had not experienced AERD, suggesting that aspirin challenge tests are critical for diagnosis [36]. The confirmative diagnosis for AERD can definitely be established by aspirin challenge [1–3, 12, 32, 36–39]. Patients receive increasing doses of aspirin during the challenges. There are four routes of provocation challenge: (1) oral, (2) bronchial inhalation, (3) nasal inhalation, (4) and intravenous [36–41]. In Europe, inhalation, nasal, or oral challenge are used; in the United States oral aspirin challenge is performed. The challenge test should be performed when asthma is stable (i.e., forced expiratory volume in the first second of expiration (FEV1) is >70% of expected value, with a variability of <10%). Increasing challenge doses of aspirin are administered (oral: ~20 to 500 mg, inhalation: ~0.2 to 182 mg cumulative dose, or lower, depending on the route of administration and severity of reported symptoms), spaced by 1.5 to 2 hours (or shorter). Nasal obstruction is measured by rhinomanometry, acoustic rhinomanometry, and/or peak nasal inspiratory flow; bronchial obstruction by FEV1. Challenges are interrupted, if a decrease of FEV1 ≥20% of baseline is measured [28, 42–45].

The challenge procedure bears the risk of severe asthma exacerbations and should be done only by trained specialists with availability of equipment and medication for the treatment of acute asthma attacks if they develop. Hypersensitivity emerged in general within 1 to 4 hours after intake of NSAIDs but may occur within minutes or takes up to 24 hours. Life-threatening reactions may occur in some patients, especially those with AERD [1–3, 28, 39–42, 44–47]. Therefore, NSAID challenge must be performed in a specialized hospital under supervision of the patient by skilled health professionals. These require proper emergency equipment, observation, and follow-up care. A brief algorithm of the diagnostic procedure is given in Table 2.

Challenge tests will fail if AERD is still not thoroughly distinctive or provocation is precluded on ethical grounds (e.g., pregnancy, children of young age), unstable asthma, asthma nonresponsive to corticosteroids, patients on *β*-blocker, anatomical alterations (e.g., massive nasal polyps), missing compliance of the patient (e.g., asthmatic experiences and therefore fear of life threatening symptoms), unavailability of specific technical and/or medical equipment (e.g., measurement of respiratory function, appropriate emergency unit), or inadequately trained staff [1, 38]. Furthermore, long-term developments such as CRS with and without polyps or gastrointestinal complications cannot be adequately followed [1] or if a prognostic goal has to be considered in patients without typical aspirin-exacerbated respiratory symptoms. In addition, oral challenge tests ruled out hypersensitivity in 50% of the patients otherwise characterised by NSAID hypersensitivity [48]. Skin test responses are typically negative. Medical history revealed best positive and negative predictive values in comparison to oral challenges, but high false-positive and negative rates were also reported [49]. In such cases *in vitro* tests might be an useful option. During the last decades several *in vitro* tests had been developed. The most promising *in vitro* tests are analysing peripheral blood leukocytes (PBLs) focusing on genes, receptors, and metabolites of the eicosanoid cascade. The characteristics and relevance for supporting the diagnosis of AERD are very recently reviewed by Schäfer and Maune [7].

The diagnosis of AERD is crucial for the onset of an appropriate therapy, but aspirin sensitivity usually is diagnosed very late during the chronological sequence of the disease. As outlined above, aspirin sensitivity might become obvious in several organs until all symptoms of AERD will have been developed (for more details, see [1]). Therefore, a full medical history placing special attention to the existence of respiratory symptoms associated to AERD is essential for an early diagnosis (see Table 1).

## 5. Molecular Pathogenesis

The molecular pathogenesis of AERD has only been partially elucidated. There are several theories that try to explain AERD. The theories trying to explain AERD include (i) alteration of the arachidonic metabolism and its receptors/enzymes, (ii) release of inflammatory mediators and cytokines, and (iii) microorganisms such as virus and bacteria.

Driven by the known inhibitory action of NSAIDs on a subset of the eicosanoid pathway, that is, the cyclooxygenase pathway, Szczeklik and colleagues speculated in an early paper in 1975 that the cyclooxygenases and their metabolic products, mainly prostaglandin E (PGE), reflect a central modulating role in patients classified by AERD [50]. A decade later he presented his theory, that a viral respiratory infection may be an inciting event that starts the inflammatory processes that lead to respiratory inflammation and AERD in genetically susceptible individuals [51]. The bronchoprotective effects of PGE2 were confirmed by Pavord and Tattersfield in 1992 [52], and elevated peptido leukotrienes (pLT) in nasal polyps were documented in 1996 by Baenkler and colleagues [53]. The interdependence of the pathways of cyclooxygenase and 5-lipoxygenase elucidated and exemplified first in 1999 by Schäfer and colleagues. They demonstrated that a profile of eicosanoids (i.e., PGE2 and pLT) is specific for AERD and might play a role in the pathogenesis of aspirin exacerbated asthma. Furthermore, an altered profile of expression, synthesis, and metabolic activity of receptors and enzymes was hypothesised [42]. Subsequent studies confirmed this concept of an AERD-specific eicosanoid pattern investigating the synthesis of eicosanoid mediators as well as the expression of receptors and enzymes in nasal secretion, urinary excretion, polypous, mucosal, and bronchial tissues in respect to the cyclooxygenase and lipoxygenase pathways [54–57]. A concept of pathogenic mechanisms, focusing on the imbalance of eicosanoid pathway was outlined recently [7]. To adumbrate this complex concept in brief, PGE as well as the corresponding receptors and enzymes are diminished even before NSAID challenge, whereas the pLT and the corresponding receptors and selected enzymes are elevated. This imbalance is potentiated upon intake of NSAIDs (for further details, see [7] and references therein).

In addition, it was shown that T cells, cocultured with parainfluenza, repiratory syncytial virus, or rhinovirus produced proliferation of CD3+ and CDR4+ cells and released cytokines that attract eosinophils [58], supporting the theory of Szczeklik [51]. Recent studies also have implicated interleukin-10 and tumor growth factor-*β*1 polymorphism through gene interaction in AERD and rhinosinusitis [59], and cyclooxygenases are modulated by cytokines [7]. Patients suffering from asthma and aspirin sensitivity as well as expressing the HLA A1/B8 phenotype have a higher incidence of AERD [60]. Elevated release of inflammatory mediators, for example, tryptase, histamine, ECP, IL-5, GM-CSF, RANTES, and eotaxin predominantly by mast cells and eosinophils, were described in patients suffering from CRSwNP and aspirin sensitivity [61–71]. The overproduction of IL-5 might intensify eosinophilic inflammation in aspirin-sensitive patients [72] and is correlated to increased levels of IgE and *Staphylococcus aureus* enterotoxins(SAE) present in nasal polyp tissue but is not specific for patients suffering from CRSwNP and aspirin sensitivity [73].

In summary, there is a complex network of molecular pathomechanisms and transcellular metabolism of eicosanoids which are implicated in the pathogenesis of CRSwNP and aspirin sensitivity [7, 66, 67, 73, 74].

## 6. Therapies

The treatment of CRS depends on the stage and extent of the disease. Several reviews and meta-analyses during the last decade point to slight differences in the pathogenesis of CRSwNP in association with aspirin intolerance compared to CRS. Therefore, we will review therapeutic approaches, which will specifically address this issue. For more details, please refer to a recent review by Alobid and Mullol [75].

### 6.1. Aspirin Desensitization

Aspirin desensitization might be beneficial for some patients classified with AERD demonstrating upper and lower airway inflammation. Aspirin desensitization is recommended for those AERD individuals with corticoid-dependent asthma or those requiring daily NSAID therapy for other medical reasons, for example, coronary artery diseases or chronic arthritis. Oral administration may reveal definitive improvements in both lower and upper airways in most patients with aspirin sensitivity [76]. The precise mechanism by desensitization in aspirin therapy is unclear. However, the synthesis of pLT by PBLs and nasal mucosa was reduced after desensitization [42, 77]. Another study demonstrated decreased bronchial responsiveness to inhaled leukotriene E4 on the day of desensitization therapy [78]. Modulation of further intracellular biochemical parameters might be another molecular mechanism (for more details on suggested mechanisms, see [7, 42]).

Focusing on the clinical symptoms, long-term aspirin desensitization (1 to 6 years) reduced significantly the use of oral corticoid treatment for asthma, the dosage of nasal corticosteroids, the numbers of sinus infections, sinus surgery per year, and hospitalization and also improved olfaction [3]. Furthermore, the rate of recurrence of nasal polyp in patients undergoing desensitization after one and six years was 6.9% and 65%, whereas without aspirin desensitization the rate was 51.3% and 93.5%, respectively [79]. Intranasal aspirin treatment in patients with bilateral CRSwNP resulted in delayed polyps recurrence, and 8 of 16 patients remained without symptoms for 15 months. The clinical outcome was significantly better than that from those treated with corticosteroids for recurrence prevention. Furthermore, endoscopy and acoustic rhinomanometry indicated a lower polyp size on the aspirin-treated nostril [80]. A double-blind, randomized, placebo-controlled trial revealed no effect on nasal airway using 16 mg of intranasal aspirin every 48 h after 6 months of treatment, but significantly decreased expression of the cys-LT1 receptor in the turbinate mucosa of aspirin treated patients [81]. Intranasal lysine-aspirin (up to 50 mg/d) in addition to routine therapy reduced polyp size without adverse effects in the lower airways [82]. Therefore, currently there is level Ib of evidence with recommendation A to use aspirin desensitization in aspirin-sensitive patients, although the evidence and recommendation to treat CRSwNP patients is still low [75].

### 6.2. Leukotriene Modifier Drugs

Leukotriene modifier drugs interrupt the leukotriene pathway and have an established impact in the therapy of asthma and allergic rhinitis. Two classes of leukotriene modifier drugs have been approved for asthma treatment: the cysteinyl leukotriene 1 receptor antagonist (montelukast, pranlukast, zafilrukast) and the 5- lipoxagenase (5LO-) inhibitor (zileuton). Both have been widely prescribed for symptom control of the upper and lower airways of patients classified by AERD [83–85].

If added to standard medication (including steroids) of patients suffering from CRS with or without nasal polyps, leukotriene modifier drugs will result in an overall improvement in nasal symptoms scores by 72%, thereby producing side effects in 11% [86]. Patients suffering from CRSwNP and treated with montelukast revealed an improvement only in some symptom scores and health-related quality-of-life parameters, whereas the nasal polyp scores and the ECP levels were not significantly altered [87, 88]. A subjective improvement in nasal symptoms was documented in 64% of aspirin-tolerant and in 50% of aspirin-sensitive patients. As significant improvements were observed only in aspirin-tolerant patients, the selective role of anti-leukotrienes in patients suffering from CRS with aspirin sensitivity was questioned [89]. Sinus symptoms improved in 60% of the patients treated with antileukotrienes, and an overall benefit was seen in 80% of patients suffering from Samter's triad [90]. Montelukast in addition to steroids significantly reduced headache, facial pain, and sneezing after eight weeks of treatment. However, no significant effect were observed on the overall symptom score, nasal blockage, hyposmia, or nasal discharge [88]. Reduced eosinophilic inflammation, viability, and cytokine production in nasal polyps following montelukast therapy was described [91].

Postoperative treatment schemes with leukotriene inhibitor revealed similar results, and no significant differences were found one year after surgery [92]. Another study investigating the postoperative effects of montelukast and intranasal mometasone medication in patients suffering from CRSwNP revealed most likely complementary results. Both treatments caused a significant reduction in the SNOT-22 scores and in the rate of nasal polyps, but only a marginal advantage of montelukast [93].

These findings point to a possible role of leukotriene modifier drugs in the treatment of specific subpopulations, but significant scientific evidence is still lacking. Therefore, leukotriene modifier drugs reveal a limited level of efficacy (III) and have a low degree of recommendation (C) in patients suffering from CRSwNP [75].

### 6.3. Surgery

Sinus surgery is recommended when medical treatment fails. Therefore, surgical procedures are often viewed as adjunctive to medical therapy [64, 94]. Additionally, medical treatment should be continued after surgery.

The greatest review on sinus surgery with 11,147 patients has been published by Dalziel et al. who screened 444 articles and evaluated 33 articles published between 1978 and 2001 [95]. In 75–95% of the cases patients consistently evaluated their symptoms to be “improved” or “greatly improved.” Additionally, surgical procedures with 1.4% overall complications for functional endoscopic sinus surgery (FESS) compared with 0.8% for conventional procedures proved to be safe.

Approximately two-thirds of the 3,128 patients participating in the National Comparative Audit suffered from CRSwNP but had no differences in clinical parameters, drug use, or general health when compared to CRSw/oNP [96]. Irrespective of the extent of surgery in the whole group of patients, a clinically significant improvement in SNOT-22 scores was demonstrated up to 36 months postoperatively. Interestingly, patients with CRSwNP benefited more from surgery than those without polyps.

Sinus surgery also significantly improved lung function and reduced systemic steroid use in patients with CRSwNP and concomitant asthma, whereas this was not the case in aspirin-sensitive asthma patients [97]. However, nasal breathing and quality of life improved in most patients.

The most recent prospective study investigated the effects of sinus surgery as well as fluticasone propionate nasal drops 400 *μ*g twice daily on nasal and lower airway parameters in asthmatics with CRSwNP. FESS significantly improved mean asthma symptom scores and daily peak expiratory flow rate (PEFR) and all nasal parameters, including subjective and objective olfaction tests. FESS improved nasal and asthma symptoms in patients with NP [98].

Taken together, sinus surgery has been proven to be an effective tool to treat CRSwNP after first-line medical therapies.

## 7. Future Attempts

### 7.1. Antibodies

#### 7.1.1. Anti-IgE/Anti-IL-5

Patients with CRSwNP had higher total IL-5 and IgE levels in nasal secretions, nasal polyp homogenisates, and blood serum than in controls. Anti-IgE or anti-IL-5 antibody only showed minimal beneficial effects on symptoms, eosinophilia and peak nasal inspiratory flow (PNIF) [99–104]. Based on current data, the evidence for efficacy of available anti-IgE and anti-IL-5 antibodies on the market is very low, and more studies are needed in order to recommend their use in the treatment of CRSwNP patients [75].

### 7.2. *Staphylococcus aureus* Enterotoxins

Nasal colonization by *Staphylococcus aureus* is a frequent event in CRSwNP patients. Accordingly, when specific IgE directed against *Staphylococcus aureus* enterotoxins (SAE) was found in nasal polyp tissue homogenates for the first time [23], a new paradigm was proposed, which indicated that these superantigens may be involved in the pathogenesis of eosinophilic CRSwNP [105]. By their superantigenic activity, enterotoxins may activate inflammatory cells in an antigen-nonspecific way. Indeed, nasal application of *Staphylococcus aureus* enterotoxin B is capable of aggravating experimental allergic asthma [86].

Interestingly, an increased colonization rate of *Staphylococcus aureus* and IgE to SAEs was reported in nasal polyps, specifically in subjects with asthma and AERD [87]. Thereby, IgE to SAEs was also coincident with higher levels of IL-5, eotaxin and eosinophil cationic protein which are known to potentiate and prolong the eosinophilic inflammation leading to polyps' development. Additionally, the presence of IgE against SAEs in CRSwNP correlated with an increased number of T cells expressing the TCR *β*-chain variable region known to be induced by microbial superantigens giving a link to the clinical importance of SAE IgE in polyps [88].

Moreover, new insights were gained into the modulatory effects of *Staphylococcus aureus* enterotoxin B (SAEB) on nasal polyp tissue [89]. SAE directed the mucosal inflammation to a Th2-driven pattern contributing to persistent inflammation by suppression of Treg lymphocytes [89]. Interestingly, SAE might only locally activate B cells, because a significant increase of local IgE antibodies can be observed in polyp patients, while independent of serum IgE levels of the same patients [23].

Nasal polyps typically show upregulation of proinflammatory pLTs and downregulation of PGE2 (see prior section for more details). In tissue of CRSwNP patients with an immune response to SAE, the production of pLTs, LTB4 and LXA4 is further upregulated [90], while SAEB significantly downregulated PGE2, COX2, and prostanoid receptor EP2 mRNA expression in fibroblasts, pointing to a direct role of SAE in regulating eicosanoids as a possible mechanism of SAE inflammatory reaction [88].

## 8. Conclusions

The latest nomenclature of AERD considers the obvious pathogenic association of chronic rhinosinusitis with/without polyps and asthma following the exposure to NSAID, which is usually last diagnosed. AERD often reveal moderate-to-severe phenotypes. Especially, the coexistence of CRSwNP elicits a more severe clinical appearance characterized by a prolonged and fatal course leading to a higher prevalence of recurrences and a coincidence with other atopic diseases. However, diagnosis in these patients is challenging despite the availability of various techniques.

AERDs are a distinct clinical entity characterized by acute NSAID triggered respiratory reactions like chronic hyperplastic rhinosinusitis with/without eosinophilia, formation of nasal polyps, or asthma. Significant morbidity and even mortality, particularly if higher doses of NSAIDs are ingested, may occur, if AERD is not recognized and appropriately treated. Concerned patients should be educated regarding NSAIDs and their avoidance preventing life-threatening asthma attacks.

The standard procedure confirming the diagnosis of AERD is still *in vivo* aspirin challenge, completed by latest blood testing, for example, in patients where *in vivo* provocation tests are contraindicated, including patients with unstable asthma, asthma nonresponsive to corticosteroid therapy, patients with *β*-blockers, and patients with potential compliance problems. Future areas of investigation should focus on the identification of further biomarkers improving early diagnosis using various diagnostic techniques.

The treatment of CRS and nasal polyps is essential to effectively control asthma and to prevent secondary infections. An adjusted medication, including aspirin desensitization, will have a positive impact on course of the disease and the patients' quality of life.

Moreover, children of parents with AERD suffer from CRSwNP and rhinosinusitis more often than children of healthy parents. This might point to a genetic background in terms of polymorphisms of COX-1, COX-2, 5-LO, and/or 15-LO pathways and/or receptors which need to be elucidated.

Our current knowledge on AERD focuses on a pathogenic concept based on decreased PGE2 and increased levels of pLT associated with overexpression of LTC4-synthase, which is accompanied by an altered transcellular metabolism of mediators of the eicosanoid cascade. This also involves local production of IgE directed to *Staphylococcus aureus* enterotoxins and overproduction of IL-5. There is a clear need to understand the implication of the metabolites of the eicosanoid cascade, their receptors, enzymes, and genes, the physiological and pathological impact of microbes, as well as the local and systemic function of dendritic cells, mast cells, and Treg cells.

The therapeutic interventions concerning the treatment of patients classified by AERD should include aspirin desensitization and medication with leukotriene modifier drugs being the most promising drugs at the moment. After failure of conservative treatment and/or in case of a severe phenotype, functional sinus surgery is an important option completed by further conservative therapies. Finally, therapeutic approaches treating *Staphylococcus aureus* and SAE effects by antibiotics or appropriate vaccination are promising.

## Figures and Tables

**Table 1:** Organ manifestation of symptoms in AERD. The classification of AERD is based on the clinical picture and becomes obvious in diverse organs at different times in the course until all symptoms of AERD will have been developed. Accurately timed diagnosis of AERD is a major challenge in patients suffering from CRS with as well as without nasal polyps and/or bronchial asthma. But also individuals without known underlying airway-related diseases have to be considered as aspirin sensitive. Thoroughly taken medical history and scrutinising the patient's organ manifestation remain a fundamental challenge. Some of the most prominent symptoms associated with AERD are summarised (without the claim of being complete) for the identification of early indicators of AERD.

Organ manifestation	Symptoms
	(i) Rhinosinusitis without nasal polyps
	(ii) Rhinosinusitis with nasal polyps
Airways	(iii) Dyspnoea
	(iv) Bronchial asthma
	(v) Laryngeal oedema

Skin	(i) Urticaria
	(ii) Angioedema

	(i) Vomiting
	(ii) Diarrhoea
Gastrointestinal tract	(iii) Dyspepsia
	(iv) Gastric bleeding
	(v) Peptic ulcer disease
	(vi) Intestinal ulcer

	(i) Cardiovascular diseases
Other organs	(ii) Anaphylaxis
	(iii) Sepsis
	(iv) Tinnitus

**Table 2:** Proposed algorithm of diagnosis of AERD. The diagnosis of AERD is a major challenge in patients suffering from CRS with/without nasal polyps, bronchial asthma, and/or unknown underlying diseases. The diagnostic approach of AERD is based on the clinical picture. This might be supported by imaging as well as in vitro techniques. The confirmative diagnosis for AERD is definitely established by aspirin challenge following increasing doses of aspirin. The routes of administration are (1) oral, (2) bronchial inhalation, (3) nasal inhalation, and (4) intravenous. Nasal or bronchial obstruction has to be monitored adequately. Provocation must be performed only when asthma is stable and is precluded on ethical grounds, unstable asthma, asthma nonresponsive to corticoids, or patients on *β*-blockers. Aspirin challenge tests should be performed by trained specialist in centres with the availability of adequate equipment and medication for emergency.

Diagnostic procedure of AERD	
Prior to aspirin challenge	

(1) Medical history	(i) Individual history
	(ii) Family history

(2) Severity of symptoms (suspected from historical reactions)	(i) No
	(ii) Mild
	(iii) Moderate
	(iv) Severe

(3) Class of NSAID	(i) Strong COX-1 inhibitors
	(ii) Poor COX-1 inhibitors
	(iii) Preferentially COX-2 inhibitors
	(iv) Selective COX inhibitors

(4) Physical examination (a) Localisation of symptoms (b) Stable asthma	(i) Airways
	(ii) Skin
	(iii) Gastrointestinal tract
	(iv) Other organs
	(v) FEV1 >70% and with 10% of best prior value

(5) Medication (a) Drug responsiveness (b) Actual medication	(i) Asthma responsiveness to corticoids
	(ii) Systemic/topic corticoids
	(iii) *β*-blockers
	(iv) Antihistamines
	(v) Others

Patient selection for aspirin challenge	

(1) Suspected reactions	(i) Mild-to-moderate prior historical reactions
(2) Responsiveness to drugs	(ii) Responsiveness to corticoids, leukotriene modifiers, *β*-blockers
(3) Anatomical alterations	(iii) No aggressive polyp formation
(4) Compliance of patient	(iv) In need of daily aspirin
(5) Pretreatment	(v) Continuing of all medications for upper and lower airways, including inhaled an intranasal corticosteroids
	(vi) Leukotriene modifier drug 2–4 weeks prior to (in case of safety reasons)

Aspirin challenge	

*In vivo* provocation according to an appropriate protocol	(i) Determination of airway stability (FEV1 >70%, 10% variability, every 1–3 h)
	(ii) Discontinue antihistamines 48 h before challenge

*In vitro* challenge, in case of	(i) Unstable asthma
	(ii) Unresponsiveness to corticoids
	(iii) Anatomical alterations
	(iv) Ethical grounds
	(v) Unavailability of technical and/or medical equipment
	(vi) In cases of non-airway-related symptoms and those not becoming obvious upon *in vivo* aspirin challenge

Treatment of aspirin-induced reactions	

Ocular Nasal Laryngeal Bronchial Gastrointestinal Urticaria/angioedema Hypotension	(i) Topical antihistamines
	(ii) (Oral) antihistamines or diphenhydramine, topical decongestant
	(iii) Racemic epinephrine nebulization
	(iv) Inhalation of *β*-agonist every 5 minutes until confortable
	(v) Empting
	(vi) Intravenous ranitidine
	(vii) Intravenous diphenhydramine
	(viii) Epinephrine administered intramuscularly
